# Neutrophil Percentage-to-Albumin Ratio as a Prognostic and Predictive Biomarker in Non-Metastatic Breast Cancer Treated with Neoadjuvant Chemotherapy: Findings from a Retrospective Cohort

**DOI:** 10.3390/diagnostics16070998

**Published:** 2026-03-26

**Authors:** Mahmut Uçar, Mukaddes Yılmaz, Eda Erdiş, Birsen Yücel

**Affiliations:** 1 Department of Medical Oncology, Sivas Cumhuriyet University, Sivas 58140, Turkey; ylmzmukaddes@gmail.com; 2 Department of Radiation Oncology, Sivas Cumhuriyet University, Sivas 58140, Turkey; dr.erdiseda@gmail.com (E.E.); yucelbirsen@yahoo.com (B.Y.)

**Keywords:** breast cancer, neutrophil percentage-to-albumin ratio (NPAR), neoadjuvant chemotherapy

## Abstract

**Background/Objectives**: This study aimed to investigate the prognostic and predictive significance of the pretreatment neutrophil percentage-to-albumin ratio (NPAR) in patients with non-metastatic breast cancer. NPAR is a composite biomarker reflecting both systemic inflammatory activity and nutritional status. Its association with treatment response and survival outcomes in patients receiving neoadjuvant chemotherapy was evaluated. **Methods**: This retrospective observational study included 194 patients diagnosed with non-metastatic breast cancer who underwent neoadjuvant chemotherapy between 2004 and 2024. Receiver operating characteristic (ROC) curve analysis was used to determine the optimal NPAR cut-off value. Patients were categorized into low-NPAR (*n* = 150) and high-NPAR (*n* = 44) groups. **Results**: Clinicopathological characteristics were comparable between the groups. However, patients with elevated NPAR values demonstrated poorer treatment responses. The objective response rate was significantly lower in the high-NPAR group compared to the low-NPAR group (70% vs. 87%). In addition, progressive disease occurred more frequently in patients with high NPAR values (16% vs. 5%). Survival analysis revealed markedly worse outcomes among patients with elevated NPAR. Multivariate Cox regression analysis confirmed high NPAR as an independent predictor of reduced overall survival (HR: 3.79; 95% CI: 1.68–8.80). **Conclusions**: Elevated pretreatment NPAR values are associated with inferior response to neoadjuvant chemotherapy and unfavorable long-term survival outcomes. NPAR may serve as a simple and cost-effective biomarker for risk stratification and could assist clinicians in identifying patients who may benefit from more individualized therapeutic strategies.

## 1. Introduction

Neoadjuvant chemotherapy (NAC) has become an important component of treatment strategies for breast cancer. Clinical evidence indicates that NAC provides survival outcomes comparable to those achieved with adjuvant therapy while offering additional benefits such as tumor downstaging and increased feasibility of breast-conserving surgery [1]. Moreover, NAC enables clinicians to directly evaluate tumor sensitivity to systemic therapy, thereby providing valuable prognostic information [2]. Achieving a pathological complete response (pCR) after neoadjuvant treatment has been consistently associated with improved long-term outcomes, including higher survival rates [3,4]. Therefore, identifying reliable biomarkers capable of predicting treatment response and disease prognosis remains an important objective in breast cancer research.

Accumulating evidence suggests that chronic systemic inflammation plays a critical role in tumor development and progression [5]. The inflammatory microenvironment influences tumor growth, angiogenesis, immune regulation, and metastatic potential. Accordingly, several inflammation-based biomarkers have been investigated as prognostic indicators in oncology. A number of systemic inflammatory markers have been investigated as prognostic indicators in breast cancer, such as the neutrophil-to-lymphocyte ratio, derived neutrophil-to-lymphocyte ratio, lymphocyte-to-monocyte ratio, platelet-to-lymphocyte ratio, and the pan-immune-inflammation value [6,7].

In addition to inflammatory parameters, nutritional status has also been recognized as an important determinant of cancer prognosis. Various composite indices reflecting nutritional and inflammatory status have been proposed, including the geriatric nutritional risk index, the hemoglobin–albumin–lymphocyte–platelet score, and the neutrophil percentage-to-albumin ratio [8,9,10]. Among these markers, NPAR has recently attracted increasing attention because it integrates two biologically relevant components: neutrophil percentage and serum albumin concentration [11].

Neutrophils represent an important component of innate immunity and are actively involved in inflammatory responses. They exert their effects through multiple mechanisms, including the secretion of cytokines, oxidative burst activity, and activation of signaling pathways such as interleukin-6, tumor necrosis factor-α, and granulocyte-colony-stimulating factor [12,13]. Elevated levels of circulating neutrophils have been associated with adverse clinical outcomes in a range of conditions, including cardiovascular diseases, infections, and different types of cancer [14,15].

Serum albumin, the most abundant plasma protein synthesized in hepatocytes, functions not only as a carrier molecule but also as an important regulator of antioxidant activity, endothelial stability, and inflammatory balance [16,17]. Importantly, albumin is considered a negative acute-phase reactant, as its synthesis decreases during inflammatory conditions due to cytokine-mediated suppression [17,18].

Because NPAR combines neutrophil percentage and serum albumin levels, it reflects both inflammatory activation and impaired nutritional or metabolic status. This dual representation makes NPAR a promising biomarker for evaluating systemic physiological stress in patients with cancer. Furthermore, NPAR can be easily calculated from routine laboratory tests, making it an accessible and inexpensive clinical parameter [19].

Although previous research has explored the relationship between NPAR and breast cancer risk or prognosis, data regarding its predictive relevance in patients with non-metastatic breast cancer undergoing neoadjuvant chemotherapy remain scarce. We hypothesized that higher baseline NPAR values may be linked to resistance to chemotherapy and could function as an independent indicator of adverse long-term outcomes, particularly decreased overall survival and disease-free survival among patients with non-metastatic breast cancer. Accordingly, the present study was designed to assess the prognostic and predictive role of pretreatment NPAR levels in individuals receiving neoadjuvant therapy for non-metastatic breast cancer.

## 2. Materials and Methods

### 2.1. Study Design

This research was performed as a retrospective cohort study with an observational design. The study protocol received approval from the Sivas Cumhuriyet University Non-Invasive Clinical Research Ethics Committee (2026-01/16, 08.01.2026). The investigation adhered to the ethical standards established in the Declaration of Helsinki. As the analysis involved previously collected and anonymized clinical data, obtaining written informed consent from participants was not necessary.

### 2.2. Study Population

Female patients aged 18 years or older who were diagnosed with breast cancer and treated at Sivas Cumhuriyet University between January 2004 and December 2024 were screened. During this period, approximately 2267 patients with breast cancer were treated, of whom 578 received neoadjuvant chemotherapy.

Among these individuals, 194 patients met all predefined inclusion criteria and had complete clinical, pathological, and laboratory data available for analysis. Patients were excluded if they had active infections, autoimmune diseases, corticosteroid use, severe hepatic or renal dysfunction affecting albumin levels, metastatic disease at diagnosis, or missing data required for NPAR calculation or survival analysis.

### 2.3. Data Collection

Demographic characteristics were collected from electronic medical records. Tumor markers at diagnosis, including carcinoembryonic antigen and cancer antigen 15-3, were also recorded. The collected clinicopathological data also comprised tumor size, T and N stages, histological subtype, tumor grade, Ki-67 proliferation index, ER, PR, and HER2 receptor status, presence of extracapsular extension (ECE), perineural invasion (PNI), lymphovascular invasion (LVI), as well as details related to treatment.

Breast cancer cases were diagnosed and staged based on the 8th edition of the AJCC Cancer Staging Manual [20]. Histological classification was performed according to the World Health Organization (WHO) guidelines for breast tumors [21], while tumor grade was evaluated using the modified Scarff–Bloom–Richardson grading system [22].

Tumors were classified as ER- or PR-positive when nuclear immunohistochemical staining was observed in at least 1% of tumor cells [1]. HER2 status was determined using both immunohistochemistry and fluorescence in situ hybridization (FISH) analyses. Cases demonstrating moderate (2+) or strong (3+) immunohistochemical staining, in conjunction with confirmatory FISH results, were categorized as HER2-positive.

The Ki-67 proliferation index was evaluated by immunohistochemical staining of tumor tissue samples obtained from formalin-fixed, paraffin-embedded specimens as part of routine pathological assessment. The Ki-67 index was defined as the percentage of tumor cell nuclei showing positive staining among the total number of tumor cells counted.

In the present study, a cut-off value of 15% was used to categorize tumors into low and high proliferative groups. This threshold was selected based on previous studies and international consensus recommendations indicating that Ki-67 values around 14–15% are commonly used to distinguish luminal A-like from luminal B-like breast cancer subtypes and to stratify tumor proliferative activity [23,24,25].

Molecular classification of breast cancer was performed in accordance with the St. Gallen International Expert Consensus recommendations [25]. Tumors positive for ER and PR but negative for HER2 were assigned to the luminal A subtype. Tumors with ER or PR positivity, absence of HER2 expression, and Ki-67 > 14 were categorized as luminal B. Patients with ER and/or PR positivity accompanied by HER2 overexpression were classified as the luminal B HER2 subtype. Cases demonstrating HER2 positivity without hormone receptor expression were defined as HER2-enriched, while tumors negative for ER, PR, and HER2 were categorized as triple-negative breast cancer. Categorization of pathological features and breast cancer subtypes were given in Table S1.

### 2.4. Assessment of Treatment Response

Treatment response was evaluated according to the AJCC 8th edition criteria. Assessment was based on pathological staging in addition to clinical and radiological evaluations.

Partial response (PR) was considered when the tumor exhibited a substantial reduction in size, typically defined as a decrease of ≥30% in the longest tumor diameter relative to pretreatment measurements.

Stable disease (SD) describes situations in which tumor size remained relatively unchanged, without meeting the criteria for either partial response or progression.

Progressive disease (PD) was defined as an enlargement of the tumor by at least 20% or the development of new lesions during therapy.

### 2.5. Treatment and Follow-Up

Anthracycline- and taxane-based chemotherapy regimens were used as the standard neoadjuvant treatment approach in the majority of patients. Patients with HER2-positive disease additionally received anti-HER2 targeted therapy in combination with chemotherapy.

When clinically indicated, adjuvant treatments included anti-HER2 therapy, endocrine therapy, or capecitabine for patients with triple-negative disease who did not achieve a pathological complete response.

All patients underwent routine follow-up visits at the Medical Oncology Department every six months. Cases of metastatic recurrence were evaluated by the institutional multidisciplinary tumor board.

Disease-free survival (DFS) was defined as the time elapsed from the initial diagnosis until the first event of recurrence, distant metastasis, or death from any cause. Overall survival (OS) was measured as the duration from diagnosis to death from any cause or the final follow-up assessment.

### 2.6. NPAR Calculation

Pretreatment peripheral blood samples were analyzed retrospectively. Neutrophil percentage values were obtained from complete blood count analyses, and serum albumin levels (g/dL) were measured at the same time point.

NPAR was calculated using the following formula:

NPAR = Neutrophil percentage/Serum albumin level

Receiver operating characteristic (ROC) curve analysis was applied to identify the optimal NPAR threshold for survival prediction. Based on this threshold, patients were subsequently classified into low-NPAR and high-NPAR groups.

### 2.7. Statistical Analysis

Data analysis was performed using SPSS software version 22.0 (IBM Corp., Armonk, NY, USA). Continuous variables following a normal distribution were expressed as mean ± standard deviation (SD), whereas non-normally distributed variables were reported as median values together with their minimum–maximum ranges. Categorical variables were summarized as frequencies (*n*) and percentages (%). Group comparisons for categorical variables were performed using Pearson’s chi-square test. When contingency tables larger than 2 × 2 demonstrated statistically significant results, Bonferroni-adjusted post hoc pairwise comparisons were performed. The Kaplan–Meier method was applied to estimate survival curves, while differences between groups were analyzed using the log-rank test. Cox proportional hazards regression analysis was subsequently performed to determine factors associated with survival outcomes. Variables that were statistically significant in univariate analysis were subsequently included in the multivariate model. Statistical significance was defined as a *p*-value ≤ 0.05.

To limit the likelihood of overfitting in the multivariate models, the inclusion of covariates was constrained based on the total number of outcome events. Additionally, potential multicollinearity among variables was assessed before model construction by examining correlations between covariates. Only variables considered clinically meaningful and statistically relevant were retained in the final models.

## 3. Results

A total of 194 patients constituted the study cohort. Following the calculation of NPAR values for each participant, receiver operating characteristic (ROC) curve analysis was performed with overall survival as the endpoint to determine the most appropriate NPAR cut-off level. The optimal value was found to be 16.32 (AUC: 0.815; 95% CI: 0.724–0.908; *p* < 0.001).

**Figure 1:**
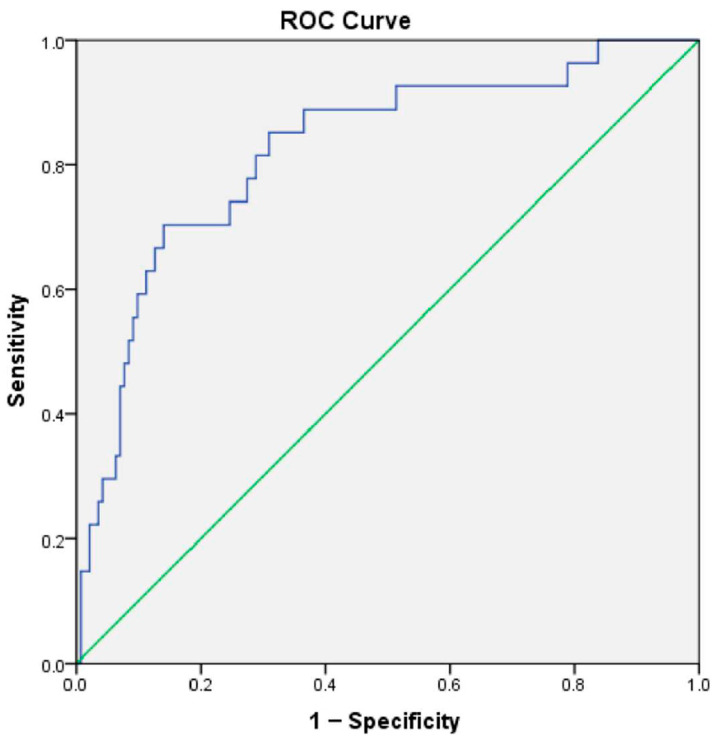
Roc Curve.

Based on the identified cut-off value, participants were stratified into two groups: 150 patients (77%) formed the low-NPAR group, while 44 patients (23%) were placed in the high-NPAR group. The ROC curve is presented in Figure 1.

### 3.1. Baseline Characteristics

The baseline demographic and clinical characteristics of the participants are shown in Table 1. The two groups were comparable with respect to age, menopausal status, and ECOG performance status, with no statistically significant differences observed. Similarly, tumor-related characteristics were largely comparable between the groups. The most common histological type was invasive ductal carcinoma, and more than half of the patients presented with stage III disease.

The distribution of T stage, N stage, and tumor grade did not differ significantly between the groups. In addition, no significant differences were observed regarding hormone receptor status (ER and PR) or HER2 expression.

Although the triple-negative subtype was proportionally more frequent in the high-NPAR group (23% vs. 10%), the difference did not reach statistical significance (*p* = 0.163). The high-NPAR group also showed higher numerical frequencies of lymphovascular invasion (LVI) and perineural invasion (PNI), but these variations were not statistically significant (*p* > 0.05).

### 3.2. Response to Neoadjuvant Chemotherapy

The comparison of treatment responses between the NPAR groups is presented in Table 2.

Pretreatment NPAR levels were significantly associated with treatment response (*p* = 0.039). Patients in the high-NPAR group demonstrated poorer responses to neoadjuvant chemotherapy compared with those in the low-NPAR group.

Specifically:

Progressive disease (PD) occurred in 16% of patients in the high-NPAR group compared with 5% in the low-NPAR group.

Stable disease (SD) was observed in 14% and 8% of patients in the high- and low-NPAR groups, respectively.

The proportion of patients achieving pathological complete response (pCR) was 34% among those in the low-NPAR group and 25% among those in the high-NPAR group.

When overall treatment response was evaluated (complete response + partial response), the response rate was significantly higher in the low-NPAR group (87%) compared to the high-NPAR group (70%) (*p* = 0.008).

### 3.3. Survival Outcomes

During the follow-up period, 27 deaths were recorded and used as events for the overall survival (OS) analysis. For disease-free survival (DFS) analysis, 34 events (recurrence, progression, or death) were observed.

**Figure 2:**
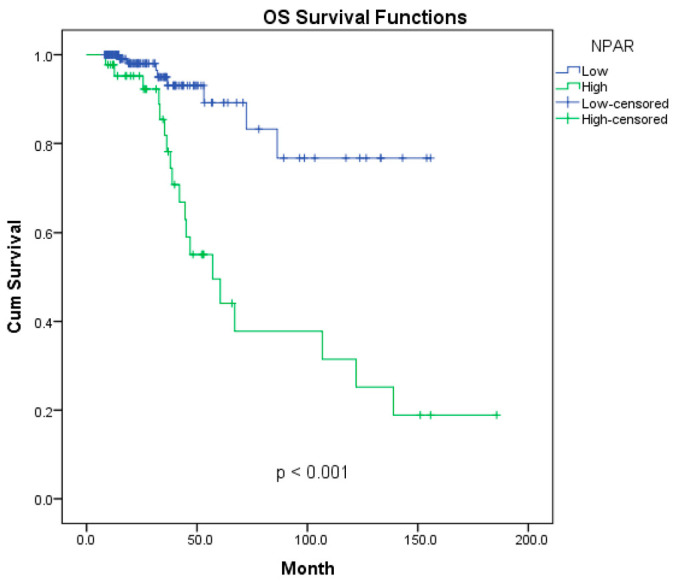
Overall survival according to NPAR status; Kaplan-Meier graph.

**Figure 3:**
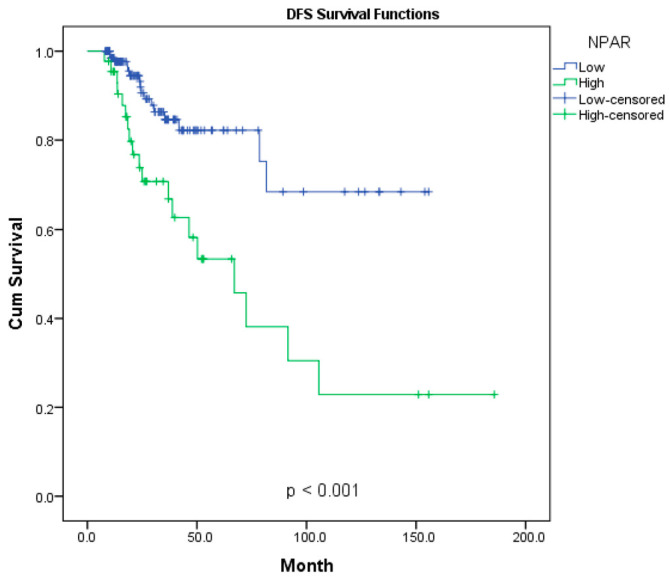
Disease free survival according to NPAR status; Kaplan-Meier graph.

The Kaplan–Meier curves for overall survival and disease-free survival are presented in Figure 2 and Figure 3. The impact of NPAR levels on long-term survival outcomes was evaluated using Kaplan–Meier analysis, which revealed significant differences between the two groups (*p* < 0.001). The 3-, 5-, and 10-year survival rates for both groups are summarized in Table 2. The 5-year OS rate was 89% in the low-NPAR group, compared to 44% in the high-NPAR group. More strikingly, while the 10-year OS rate was 77% in the low-NPAR group, it dropped to 25% in the high-NPAR group. The median OS for the high-NPAR group was determined to be 57 months. Regarding DFS analysis, the low-NPAR group exhibited a 5-year rate of 82%, whereas this rate was 38% in the high-NPAR group. At the 10-year follow-up, 69% of patients in the low-NPAR group remained disease-free, compared to only 23% in the high-NPAR group. Overall survival and disease-free survival curves are illustrated in Figure 2 and Figure 3, respectively.

Prognostic factors affecting OS are analyzed in Table 3. In the univariate Cox regression analysis, high NPAR (*p* < 0.001), failure to achieve pCR (*p* = 0.045), presence of PNI (*p* = 0.017), presence of LVI (*p* = 0.047), tumor necrosis (*p* = 0.011), presence of ECE (*p* = 0.002), elevated CEA levels (*p* = 0.025), and multicentricity (*p* = 0.047) were identified as significant prognostic parameters. According to the multivariate analysis results, high NPAR (HR: 3.79; 95% CI: 1.68–8.80; *p* = 0.002), presence of tumor necrosis (HR: 2.86; 95% CI: 1.14–7.19; *p* = 0.025), and elevated CEA levels (HR: 3.34; 95% CI: 1.34–8.32; *p* = 0.009) were determined to be independent poor prognostic factors for OS. Notably, pCR status, PNI, LVI, ECE, and multicentricity, which showed significance in the univariate analysis, did not retain their independent statistical significance in the multivariate model.

The Cox regression analysis conducted to identify prognostic factors affecting DFS is presented in Table 4. In the univariate analysis, high NPAR levels (*p* = 0.001), failure to achieve pCR (*p* = 0.016), presence of PNI (*p* = 0.024), presence of LVI (*p* = 0.024), tumor necrosis (*p* = 0.020), and ECE (*p* = 0.001) were found to be significantly associated with DFS. However, when these significant variables were included in the multivariate Cox regression model, only high NPAR remained an independent prognostic factor for DFS (HR: 2.80; 95% CI: 1.43–5.48; *p* = 0.003). Notably, pCR status and ECE, which were significant in the univariate analysis, did not reach independent statistical significance in the multivariate model (*p* > 0.050).

## 4. Discussion

The response to neoadjuvant chemotherapy in breast cancer provides important insight into the patient’s long-term prognosis. However, accurately identifying patients who are likely to develop resistance to treatment remains a major clinical challenge. Our findings indicate that the NPAR, which reflects both inflammatory activity and nutritional condition, has significant prognostic relevance in patients with non-metastatic breast cancer. Patients with elevated NPAR values exhibited reduced objective response rates, a greater risk of disease progression during therapy, and markedly poorer survival outcomes. The results of the multivariate analysis demonstrated that NPAR independently predicted both disease-free survival and overall survival.

The association between cancer progression and systemic inflammation has been well established in the literature. Neutrophils can promote tumor angiogenesis and invasion through the secretion of vascular endothelial growth factor (VEGF) and matrix metalloproteinases (MMPs), whereas serum albumin levels reflect the host’s inflammatory status and nutritional reserve [26]. Renard et al. demonstrated that nutritional and inflammatory characteristics are among the primary determinants of prognosis, independent of tumor origin or metastatic status [27]. Moreover, inflammation and nutrition are closely interconnected biological processes. Nutritional status influences inflammatory responses, while inflammation in turn affects metabolic and nutritional pathways [28].

Recent studies have increasingly emphasized the importance of identifying reliable prognostic biomarkers in breast cancer due to the heterogeneous nature of the disease and the variability in treatment response among patients. Breast cancer prognosis is influenced not only by classical clinicopathological parameters such as tumor size, nodal status, histological grade, and molecular subtype, but also by systemic biomarkers reflecting tumor–host interactions. In this context, López-González et al. reported that biomarkers related to systemic inflammation and immune-nutritional status are gaining increasing importance in clinical oncology, complementing traditional molecular markers such as ER, PR, HER2, and Ki-67 in predicting disease outcomes [29]. Similarly, Popa et al. emphasized that breast cancer represents a biologically heterogeneous disease in which multiple biological, pathological, and systemic factors contribute to prognosis and treatment response [30].

More recently, composite indices that combine inflammatory and nutritional parameters have gained attention as potential prognostic markers. Among these markers, the NPAR has recently been recognized as a promising biomarker that reflects both systemic inflammatory activity and nutritional status. Large population-based analyses have demonstrated that elevated NPAR levels are associated with increased breast cancer incidence and mortality. Su et al., in an analysis of the NHANES cohort, found that increased NPAR levels were independently correlated with both breast cancer risk and all-cause mortality, highlighting the potential role of this biomarker in reflecting systemic mechanisms associated with tumor initiation and progression [31]. Similarly, in a large population-based study of 18,726 individuals, elevated NPAR levels were found to be significantly associated with breast cancer prevalence [32]. Another analysis involving 14,211 female participants demonstrated a linear positive relationship between NPAR values and breast cancer risk [33].

One of the most notable findings of our study was the significant association between NPAR and treatment response. The significantly lower objective response rate (70% vs. 87%) and the higher incidence of progressive disease (16% vs. 5%) observed in the high-NPAR group suggest that systemic inflammation may contribute to chemotherapy resistance. In a retrospective study evaluating neoadjuvant chemotherapy protocols in patients with triple-negative breast cancer, Zhang et al. reported that high NPAR status was an independent predictor of pathological complete response [34]. Our results, however, did not reveal a statistically significant association between NPAR and pCR. This discrepancy may be explained by the multifactorial nature of pCR, which is strongly influenced by tumor biology, molecular subtype, chemotherapy regimen, and treatment duration. Therefore, it is plausible that a single systemic inflammatory marker may not independently predict pCR. In contrast, NPAR may more strongly reflect the host’s systemic inflammatory and nutritional status, thereby exerting a greater influence on long-term survival outcomes.

Several studies conducted in different malignancies have also reported that high NPAR values are associated with poor prognosis. Elevated NPAR levels have been associated with worse outcomes in colorectal cancer and prostate cancer [35,36]. In oral cavity cancer, a high preoperative NPAR value (≥16.93) was associated with significantly poorer overall and disease-free survival compared with lower values [37]. Similarly, in colorectal cancer patients, elevated NPAR levels have been shown to predict poorer overall survival and progression-free survival [35]. In patients with invasive bladder cancer receiving neoadjuvant chemotherapy followed by radical cystectomy, increased NPAR values were also associated with reduced overall and cancer-specific survival [38]. Moreover, Liu et al. showed that individuals with metastatic breast cancer had significantly higher NPAR levels than patients with non-metastatic disease, and these elevated levels were correlated with poorer overall survival outcomes [39].

Consistent with these findings, our study demonstrated a pronounced reduction in both overall survival and disease-free survival among patients with high NPAR levels. The substantial differences observed between the low- and high-NPAR groups support the hypothesis that cancer-related systemic inflammation and impaired nutritional status contribute significantly to disease progression and prognosis [40]. Our results extend previous findings by demonstrating the prognostic significance of NPAR specifically in patients with non-metastatic breast cancer receiving neoadjuvant chemotherapy.

Kaplan–Meier survival analyses in our study demonstrated significant differences between the low- and high-NPAR groups. However, Cox regression analyses provide adjusted estimates that account for potential confounding variables. Therefore, while Kaplan–Meier curves reflect unadjusted comparisons, multivariate Cox models provide a more accurate estimation of the independent prognostic contribution of NPAR. These methodological differences should be considered when interpreting the prognostic role of this biomarker.

This study has certain limitations that warrant consideration. The retrospective design may lead to potential selection bias. Additionally, because the study was carried out at a single center, the applicability of the results to other settings may be restricted. In addition, only pretreatment NPAR values were evaluated; therefore, potential changes in inflammatory markers during treatment could not be assessed. Furthermore, although patients with overt infections or systemic inflammatory diseases were excluded, albumin and neutrophil levels may still be influenced by subclinical conditions.

Another limitation is the relatively modest sample size, particularly the smaller number of patients in the high-NPAR group, which may reduce the statistical power of the analyses. Additionally, the optimal NPAR cut-off value was determined using ROC analysis within the same cohort in which its prognostic value was evaluated, which may increase the risk of overfitting. Moreover, NPAR was analyzed as a dichotomized variable based on an ROC-derived threshold, which may lead to loss of information. Therefore, the proposed cut-off value should be interpreted cautiously and requires validation in larger independent cohorts.

Despite these limitations, the present study has several strengths. To our knowledge, relatively few studies have specifically evaluated the prognostic and predictive value of NPAR in patients with non-metastatic breast cancer undergoing neoadjuvant chemotherapy. Our study included detailed clinicopathological data and long-term follow-up, allowing the comprehensive evaluation of both treatment response and survival outcomes. Furthermore, NPAR is derived from routinely available laboratory parameters, making it a simple and cost-effective biomarker with potential clinical applicability.

## 5. Conclusions

In conclusion, this study demonstrates that elevated pretreatment NPAR levels are strongly associated with poor clinical response to neoadjuvant therapy and unfavorable long-term survival outcomes (OS and DFS) in patients with non-metastatic breast cancer (nMBC). NPAR represents a valuable tool for risk stratification and the planning of personalized treatment strategies in clinical practice. It may be a rational approach to consider patients with high NPAR levels as potential candidates for more aggressive adjuvant therapies or experimental treatment protocols beyond standard care. Nevertheless, multicenter and prospective randomized trials are warranted to further validate the clinical utility of these findings.

## Figures and Tables

**Table 1:** Baseline Demographic and Clinical Characteristics.

	Low NPAR	High NPAR	*p* Value
	*n* = 150 (77%)	*n* = 44 (23%)	
Age (median), years	52 (24–82)	51 (29–81)	0.723
Menopausal status			
Premenopausal	77 (51)	18 (41)	0.224
Postmenopausal	73 (49)	26 (59)	
ECOG PS			
ECOG 0–1	150 (100)	43(98)	0.052
ECOG 2 and above	0 (0)	1 (2)	
Invasive ductal	138 (92)	40 (91)	0.800
Other	12 (8)	4 (9)	
Stage			
I	2 (1)	0 (0)	0.691
II	67 (45)	19 (43)	
III	81(54)	25 (57)	
T stage			
T1	27 (18)	6 (14)	0.147
T2	90 (60)	21 (48)	
T3	10 (7)	8 (18)	
T4	23 (15)	9 (20)	
N stage			
N0	14 (9)	7 (16)	0.466
N1	58 (39)	16 (36)	
N2–3	78 (52)	21 (48)	
Grade			
I	22 (15)	4 (9)	0.260
II	88 (59)	23 (52)	
III	40 (26)	17 (39)	
ER			
Negative	38 (25)	16 (36)	0.151
Positive	112 (75)	28 (64)	
PR			
Negative	43 (29)	19 (43)	0.069
Positive	107 (71)	25 (57)	
HER2			
Negative	90 (60)	30 (68)	0.540
Positive	59 (40)	14 (32)	
Ki-67			
<15	31 (21)	6 (17)	0.542
≥15	115 (79)	30 (83)	
Molecular BC Subtypes			
Luminal-A-Like	31 (21)	5 (11)	
Luminal B (HER2−)	45 (30)	15 (34)	0.163
Luminal B(HER2+)	40 (27)	8 (18)	
HER2-enriched BC	18 (12)	6 (14)	
Triple-negative BC	16 (10)	10 (23)	
LVI			
No	104 (69)	23 (54)	0.128
Yes	46 (31)	20 (46)	
PNI			
No	124 (83)	30 (70)	0.143
Yes	26 (17)	70 (30)	
ECE			
No	100 (67)	25 (58)	0.302
Yes	50 (33)	18 (42)	
CEA			
Normal	123 (87)	33 (79)	0.165
High	18 (13)	9 (21)	
CA 15-3			
Normal	100 (69)	26 (62)	0.390
High	45 (31)	16 (38)	

NPAR—neutrophil percentage/albumin ratio; ECOG PS—Eastern Cooperative Oncology Group performance status; T stage—tumor stage; N stage—nodal stage; LVI—lymphovascular invasion; PNI—perineural invasion; ECE—extracapsular extension; CEA—carcinoembryonic antigen; CEA normal < 5.2 ng/mL; CA 15-3—cancer antigen 15-3; CA 15-3 normal ≤ 25 U/mL.

**Table 2:** Response to Neoadjuvant Chemotherapy by NPAR Group and Survival Outcomes (Kaplan–Meier Analysis).

Response	Low NPAR	High NPAR	*p*
Progressive Disease	7 (5) ^	7 (16) ^	
Stable Disease	12 (8)	6 (14)	
Partial Response	80 (53)	20 (45)	0.039 *
Complete Response	51 (34)	11 (25)	
Objective Response			
Yes (CR + PR)	131 (87)	31 (70)	0.008 *
No (SD + PD)	19 (13)	13 (30)	
Overall Survival (OS)			
3 year (%)	95	78	
5 year (%)	89	44	<0.001 *
10 year (%)	77	25	
Median	NA	57	
Disease-Free Survival (DFS)			
3 year (%)	85	67	
5 year (%)	82	38	<0.001 *
10 year (%)	69	23	
Median	NA	57	

NPAR—neutrophil percentage/albumin ratio; CR—complete response; PR—partial response; SD—stable disease; PD—progressive disease. ^ Post hoc pairwise comparisons with Bonferroni correction; OS—overall survival; DFS—disease-free survival; NA—not applicable; * *p* < 0.05.

**Table 3:** Cox Regression Analyses for Overall Survival.

Variables	Univariate Analysis			Multivariate Analysis		
	HR	95% CI	*p* Value	HR	95% CI	*p* Value
NPAR						
Low	1			1		
High	5.21	2.27–11.97	0.000	3.79	1.68–8.80	0.002
pCR						
Yes	1			1		
No	7.751	1.04–57.41	0.045	4.87	0.64–36.67	0.124
PNI						
No	1			1		
Yes	2.54	1.18–5.50	0.017	0.73	0.24–2.21	0.582
LVI						
No	1			1		
Yes	2.21	1.01–4.85	0.047	1.18	0.40–3.49	0.760
Tumor necrosis						
No	1			1		
Yes	2.78	1.26–6.14	0.011	2.86	1.14–7.19	0.025
ECE						
No	1			1		
Yes	3.63	1.58–8.33	0.002	1.35	0.51–3.53	0.536
CEA						
Normal	1			1		
High	2.48	1.12–5.49	0.025	3.34	1.34–8.32	0.009
Multicentric						
No	1			1		
Yes	2.55	1.01–4.85	0.047	1.30	0.42–4.02	0.640

NPAR—neutrophil percentage/albumin ratio; pCR—pathologic complete response; PNI—perineural invasion; LVI—lymphovascular invasion; ECE—extracapsular extension; CEA—carcinoembryonic antigen; HR—hazard ratio; 95% CI—95% confidence interval.

**Table 4:** Cox Regression Analyses for DFS.

Variables	Univariate Analysis			Multivariate Analysis		
	HR	95% CI	*p* Value	HR	95% CI	*p* Value
NPAR						
Low	1			1		
High	3.21	1.64–6.25	0.001	2.80	1.43–5.48	0.003
pCR						
Yes	1			1		
No	5.80	1.38–24.31	0.016	3.38	0.73–15.57	0.118
PNI						
No	1			1		
Yes	2.21	1.10–4.41	0.024	0.79	0.34–1.86	0.598
LVI						
No	1			1		
Yes	2.18	1.11–4.30	0.024	0.94	0.42–2.12	0.894
Tumor necrosis						
No	1			1		
Yes	2.36	1.14–4.89	0.020	1.33	0.62–2.85	0.452
ECE						
No	1			1		
Yes	3.24	1.61–6.54	0.001	2.07	0.98–4.39	0.057

NPAR—neutrophil percentage/albumin ratio; pCR—pathologic complete response; PNI—perineural invasion; LVI—lymphovascular invasion; ECE—extracapsular extension; HR—hazard ratio; 95% CI—95% confidence interval.

## Data Availability

The raw data supporting the conclusions of this article will be made available by the authors on request.

## Supplementary Materials

The following supporting information can be downloaded at https://www.mdpi.com/article/10.3390/diagnostics16070998/s1: Table S1: Categorization of pathological features and breast cancer subtypes.

## Abbreviations

The following abbreviations are used in this manuscript:

NAC	Neoadjuvant chemotherapy
nMBC	Non-metastatic breast cancer
NPAR	Neutrophil percentage-to-albumin ratio
pCR	Pathologic complete response
OS	Overall survival
DFS	Disease-free survival
NHANES	National Health and Nutrition Examination Survey
ER	Estrogen receptor
PR	Progesterone receptor
HER2	Human epidermal growth factor receptor 2
AJCC	The American Joint Committee on Cancer
PNI	Perineural invasion
LVI	Lymphovascular invasion
ECE	Extracapsular extension
