# Supplementary material for: Neutrophil Percentage-to-Albumin Ratio as a Prognostic and Predictive Biomarker in Non-Metastatic Breast Cancer Treated with Neoadjuvant Chemotherapy: Findings from a Retrospective Cohort
Source: Diagnostics (Basel). 2026 Mar 26;16(7):998. doi: 10.3390/diagnostics16070998 (PMC13073549; doi:10.3390/diagnostics16070998)
Supplement: Supplementary file 1 [file diagnostics-16-00998-s001.zip › diagnostics-4166648-supplementary.pdf]

Table S1: Categorization of pathological features and breast cancer subtypes

Parameter	Method of evaluation	Definition used in the study
ER	Immunohistochemistry	Positive if ⩾1% tumor cell nuclei staining
PR	Immunohistochemistry	Positive if ⩾1% tumor cell nuclei staining
HER2	IHC / FISH	Classified according to ASCO/CAP guidelines
Ki-67	Immunohistochemistry	Percentage of positive tumor cells
LVI	Histopathological examination	Presence of tumor cells in lymphatic or vascular
PNI	Histopathological examination	Presence of tumor cells surrounding or invading nerve fibers
ECE	Histopathological examination	Tumor extension beyond lymph node capsule
Luminal A BC	Immunohistochemistry	ER and PR positivity and HER2 negativity Ki-67<15
Luminal B BC HER2 (-)	Immunohistochemistry	ER or PR positivity and HER2 negativity Ki-67≥15
Luminal B BC HER-2 (+)	Immunohistochemistry	ER and/or PR positivity and HER2
HER-2 enrich	Immunohistochemistry	HER2 positive
Triple negative	Immunohistochemistry	ER, PR, and HER2 negativity
